# That Bedroom

**Published:** 1881-07

**Authors:** J. S. Galloway


### THAT BEDROOM.

What about it? Well, a good many things might be said. It ought to be a place for quiet and refreshing sleep. But it is not such a place. Restless tossings with troubled dreams are there. Morning after morning finds the sleeper weary, listless, and dumpish. He wonders why it is so, and we wonder too. But our wonder is that he does not make it a matter of thought, and learn how to sleep as he should. When, anything is wrong with us, there is a cause for it. As a rule, that cause is not so obscure as to require the aid of a modern scientist, with all his jargon of incomprehensible technicalities covering his still more incomprehensible ideas, or want of ideas, to ferret it out.

To exorcise the demon of restlessness is not bedroom work alone. He who seeks the comfort of sound, refreshing sleep must properly control his habits by day as well as by night.

So much may be said about sleep in general that the bedroom is in danger of being forgotten. After all, that particular bedroom does not differ materially from many others of its kind. Twelve feet long, ten feet wide, and seven feet six inches high, it has a capacity of nine hundred cubic feet. It has a door opening into the next room, and another into the hall leading into the stairway and hall below. One large window, with sash supported by pulleys and weights affords, or ought to afford, air and light from outdoors. The fourth wall is solid. The bed stands in a corner, with the head to this wall. Two walls confine the exhaled air about the head of the sleeper. The bed rests on springs, with mattress and feathers above. The breath of the sleeper is doubly foul from late and full suppers, and from ulceration of the respiratory membrane, caused by chronic catarrh. In such a case good ventilation is more than usual a necessity. Is it attended to? Take a peep at that room. Doors closed—windows carefully closed to keep out night air. Any good work on ventilation can be consulted to ascertain how long nine hundred cubic feet of air will supply respiratory material in such a room for one occupant. And yet that is a nice bedroom, genteelly furnished. If good sleeping is not done there, the failure is less chargeable to the room than to its management. How many cases of the kind have you met with, reader!—*Phrenological Journal.*

J. S. GALLOWAY, M. D.

